# Safe Carrying of Heavy Infants Together With Hair Properties Explain Human Evolution

**DOI:** 10.3389/fpsyg.2022.854948

**Published:** 2022-05-31

**Authors:** Lia Queiroz do Amaral

**Affiliations:** Department of Applied Physics, Institute of Physics, University of São Paulo, São Paulo, Brazil

**Keywords:** infant carrying, survival of heavy infants, hair properties, nakedness, bipedalism, thermoregulation, huddle, early social structure

## Abstract

As a physicist, my scientific career was interrupted by maternity, and afterward retaken, with a parallel independent personal perspective on human evolution. My previous published contributions are reanalyzed as Hypothesis and Theory. The focus is on safe infant carrying in primates, sexual selection among Hominoidea, fur reduction in hominins, and tensile properties of hominoid hairs, justifying the necessary change to bipedal locomotion from the overwhelming selective pressure of infant survival. The Discussion starts with analysis of existing bias against acceptance of these new ideas, first with rational arguments on bias existing between Exact Sciences and Biological Sciences. A reanalysis of data on elasticity of hominoid hairs is made, based on published differences between statistical analysis of measurements in exact and inexact sciences. A table constructed from the original data on hair elasticity allows a simplified discussion, based on statistics used in Physics in the study of “known samples,” adding extra information to the available data. Published data on hair density in primates and mammals allow the conclusion that hair elastic properties might have evolved correlated to the pressure of safe carrying of heavy infants, with an upper limit of 1 kgf/cm^2^ for safe infant clinging to primate mother’s hair. The Discussion enters then on the main ideological bias, related to the resistance in the academy to the idea that bipedalism could be connected to a “female problem,” that means, that it was not a “male acquisition.” Tripedal walk, occurring naturally among African Apes carrying their newborns, unable to support themselves by ventral clinging, is the natural candidate leading to evolution of bipedal locomotion. Tripedal walk as an intermediate stage to bipedalism was in fact theoretically proposed, but ignoring its role in primate transportation by ape mothers. The Discussion proceeds to a proposal of phylogenetic evolution of Hominoids, the usual focus on the males changes to the role of females with infants, allowing an integrated view on Hominin evolution, with fur reduction and thermoregulation of the naked skin, with subcutaneous insulating fat layer. The model for earliest hominin social structures is based on huddle formation and hormonally defined rites of passage.

## Introduction

The Frontiers Research Topic “A 150 Years’ Celebration of Darwin’s Book on Human Evolution and Sexual Selection: Its Legacy and Future Prospects” included explicitly “Among other possibilities, we also encourage researchers to submit biographical comments about the significance of Darwin and his book to one’s career, and how it has impacted one’s research interests.” My article starts with biographical comments on the impact of Darwin’s Sexual Selection on me.

I came in contact with Darwin’s book (Darwin, 1871) by chance, in 1975, while walking in the street where I then lived. I crossed the street exactly in front of a bookshop, where the book was exposed in the showcase, since it was published the year before in my language, Portuguese. I decided to buy it because I was recovering from a crisis which changed completely my life, and the subject fitted exactly in what I was looking for, without even knowing it.

Since childhood, I had facility in mathematics, but difficulties with social relations, so I chose graduation in Physics and started to do scientific research still as a student, in a Nuclear Research Reactor, where I got a job immediately after graduation. I worked there for more than a decade doing hard science, and have been sent abroad for a 15 months stage in Sweden in 64/65. After my return to São Paulo, Brazil, I started to publish scientific articles, got married, completed my Master’s degree in Nuclear Science, and started to work in my Ph.D. thesis (on Molecular Physics). After getting the data, I discovered that I was pregnant, started to write the thesis, and my daughter was born (December 1972) 5 days after my thesis defense.

The next year was terrible, I had not enough time to stay with my baby, rebelled against male authoritarianism at work, and finally decided to quit my full time job, family and personal life became priority. After some months at home I was invited to start a new part time compromise, and returned to the Physics Institute of the University of São Paulo (IFUSP), giving classes and starting to work in a new research project. I succeeded in making a proposal to start a new Laboratory (X-ray Crystallography) in a new research field (Liquid Crystals). But marriage collapsed, and some months later I did have a personal breakdown, with a slow recovery.

The period from January 1974, when I left the Reactor, to August 1976, when I won a contest for tenure at IFUSP, defined a change in life perspective, together with Darwin’s book. It was completely different than what I had learnt at school, when only natural selection was mentioned in biology. I underlined all important points of Darwin’s text (715 pages), striking for me were the human skin and sexually dimorphic vocalizations, besides the evidence on animals. The idea of esthetic female choice was especially intriguing for me. I read also in Portuguese “the naked ape” (Morris, 1967). All these ideas about Biological Evolution were then mixed with studies of psychoanalysis (Freud and several authors), pedagogy (Piaget) and anthropology (Levi Strauss and Roger Bastide). In 1976 my daughter went to the kindergarten, the new Laboratory was installed at USP and I could conciliate my academic duties with time for private studies satisfying my curiosity.

For the next decades my research evolved in the interfaces physics/chemistry/biology/education at IFUSP, where I became Full Professor in 1991. My independent studies lead in parallel to three papers in Human Evolution (do Amaral, 1989, 1996; Amaral, 2008).

The next item gives a personal perspective on the development of ideas and literature on Evolution from the 19^th^ to 21^st^ centuries. After that, the section on Results presents the basic knowledge necessary for the Discussion made later on in this article.

## Perspective Since the 19th Century

The interest of Charles Darwin on human mind and instincts existed before his book on Natural Selection (Darwin, 1859), but he was very cautious and prudent in expressing these ideas. Only in his book on Sexual Selection (SS) (Darwin, 1871) he discusses explicitly our species, as one among others, and immediately after that comes his book “The expressions of the Emotions in Man and Animals” (Darwin, 1872), where he exposes more of his private life, in family photos. He considered the three books as a trilogy, and in fact his ideas prepared the modern disciplines of ethology and psychology.

It should be stressed the existing ideas in the 19^th^ century. The term “biologie,” derived from greek (Aristotle), was already defined by 1800. The term “evolution,” referred to the development of the individual embryo, and started to be used for species change.

The British philosopher and positivist scientist Herbert Spencer, a decade younger than Darwin, introduced “social Darwinism” and the concept of “struggle for existence ruled by survival of the fittest,” which appears in Darwin’s book. Spencer central ideas emphasized the social organism and the social self-consciousness of individuals (Offer, 2019). The dualism individual versus society is indeed a permanent philosophical and practical problem.

In Darwin’s time only the proposal of natural selection caused real impact, since the intellectual ambient was already prepared to absorb it. His proposal of sexual selection, particularly in relation to female choice received strong resistance, and his book on Expression of Emotions became popular, but did not receive much scientific credit.

In Darwin’s autobiography written for his family he states what was already in SS: he considered that Natural Selection needed several corrections. He was since childhood interested in collecting things, as a systematic naturalist, but also as a very ordinary boy. After his 3 years in Cambridge he joined the Beagle as a Naturalist without pay, for 5 years (1831–1836). The way this voyage defined his latter work was much discussed (Sulloway, 1982), but I prefer to stay with his own words, on pg. 119/120 of his autobiography (Darwin and Barlow, 1958):

– *My first note-book was opened in July 1837. I worked on true Baconian principles, and without any theory collected facts on a wholesale scale, more especially with respect to domesticated productions, by printed enquiries, by conversations with skillful breeders and gardeners, and by extensive reading*….
– *I soon perceived that selection was the keystone of man’s success in making useful races of animals and plants. But how selection could be applied to organisms living in a state of nature remained a mystery to me.*

Clearly, artificial selection, by breeding intentionally, was the first obvious mechanism drawing Darwin’s attention. In pg. 121 starts his telling about receiving in summer 1858 Wallace’s essay “On the Tendency of Varieties to depart indefinitely from the Original Type,” with similar ideas. He joined his ideas with Wallace’s essay, published together in the Journal of the Proceedings of the Linnean Society 1858, p. 45. He says on p. 122 of his autobiography:

– *Nevertheless our joint production excited very little attention*….
– *In September 1858 I set to work by the strong advice of Lyell and Hooker to prepare a volume on the transmutation of the species*….
– *It was published under the title of the Origin of Species, in November 1859*……

Darwin ideas on artificial selection became the first chapter on his book on natural selection. As discussed later (Brown, 2010) variation under domestication was the paradigm. Soon later Darwin published “The Variation of Animals and Plants under Domestication,” expanding that first chapter, and this book (Darwin, 1868) became one of the foundation of scientific plant breeding.

Darwin did have ideas on “heredity,” but he accepted Lamarck’s views on heredity of acquired characteristics, which was at his time the only systematic theory of biological evolution (Lamarck, 1809). In the Origin of the Species he accepts the inheritance of acquired characteristics as one of the factors contributing to evolution.

An interesting review (Jordanova, 1986) criticizes the first English translation of Lamarck’s book.

Genetics came later on and there is evidence that Gregor Mendel, younger but contemporary of Darwin, knew about Darwin, while Darwin did not know about Mendel (Fairbanks, 2020). The meaning of Mendel’s work on heredity, published in 1866, became clear only in the beginning of the 20^th^ century, when it was rediscovered (Keynes and Cox, 2008) (Simunek et al., 2011).

Regarding the following scientific progresses it should be stressed the contributions of Ronald Fisher, British mathematician, statistician and geneticist, first in his work on sexual selection as a genetic runaway process (Fisher, 1915) and later on his analysis of Mendel’s data on experimentation with pea plants (Fisher, 1936), which originated a hot debate later on (Franklin et al., 2008).

Modern Genetics paved the route for the Modern Synthesis, defined by the union of natural selection with genetics, in the period between the two Great Wars. The large amount of work of this period can be seen from a perspective of some decades later, in some specific references:

– Evolution: The Modern Synthesis (Huxley, 1942), and a Definite Edition, with a Foreword (Pigliucci and Müller, 2010).
– Genetics of the Evolutionary Process (Dobzhansky, 1970), with a Book review (Harrison, 1972).
– Origins of the Modern Synthesis: *The Evolutionary Synthesis*. Perspectives on the Unification of Biology (Mayr and Provine, 1980), with a book review (Ruse, 1981).

It should be stressed that the Modern Synthesis had two problems:

– The whole field of fetal development and embryogenesis (a direction strong in Germany) was discarded in favor of population genetics.
– Sexual selection became only objective reproduction with competition between males, and female sexuality was ignored.

From Darwin’s ideas on animal behavior emerges the work on ethology, by Konrad Lorenz and Niko Tinbergen, influencing psychology already in the 30’s. A perspective on their work can be found in a book (Burkhardt, 2005) with a review (Clucas, 2006). Lorenz and Tinbergen, together with Karl von Frisch (who worked on bees), received the Nobel Prize for Physiology and Medicine in 1973.

The revival of Darwin’s ideas on Expression of Emotions occurs only with psychologist Paul Ekman, almost hundred years after Darwin. In 1954 he begins his research on facial expression and body movements, including hand gestures, followed by non-verbal behavior and micro-expressions. He discovers universal facial expressions and publishes books and articles (Ekman, 1999, 2007, 2016). The field of Evolutionary Psychology develops, together with Emotions (Cosmides and Tooby, 2000).

In the centenary of Darwin’s book on sexual selection 11 essays were published in a book (Campbell, 1972), with a review (Williams, 1973). There the parental investment theory (Trivers, 1972) expands sexual selection and predicts that the sex that invests more in its offspring will be more selective when choosing a mate, and the less-investing sex will have intra-sexual competition for access to mates.

Detailed studies in sexual selection flourished in the last decades, both on animals and plants (Andersson, 1994; Andersson and Iwasa, 1996; Andersson and Simmons, 2006).

Regarding sexual selection among humans, the specific proposals of Darwin were retaken more recently in psychological academic papers, focusing physical attractiveness (Dixson et al., 2007, 2010, 2011; Valentova et al., 2017), based usually on attractiveness of male or female figures, eye tracking and also questionnaires to volunteers.

Regarding sexual selection among monkeys, Darwin published a very interesting article on Nature (Darwin, 1876). My first article (do Amaral, 1989) quotes the frontier literature at that time, and this article discusses again the subject.

The following item presents the results necessary to proceed in this article on Hypothesis and Theory, focusing safe carrying of heavy infants together with hair properties as a possible explanation of Human Evolution.

## Results of Interest to this Proposal

### My Previous Initial Contribution to Human Evolution

In this item I synthesize my three papers on Human Evolution (HE), mentioned in the Introduction. It must be stressed that the present focus on the subject “bipedal locomotion” is a consequence of development of objective criteria to define, from the existing bones, if a given fossil was a biped or not. Locomotion is of course important, but all anthropoids are able to bipedal walk for short periods, the point is to understand why such peculiar form of locomotion became necessary in Human Evolution.

#### Paper 1—Global View

My original proposal (do Amaral, 1989). Joins “hypothesis and theory,” with the view I had at that time. The complete text (with 60 references) is available in pdf image format. I copy the abstract and make some comments.

##### Abstract

Hindrances against bipedalism evolution are localized in obstetrical constraints, maternal mortality rates, infant birth trauma and unsafe pregnancy. Analysis of infant survival probability shows that a shift to bipedalism could occur as a necessary consequence of the process of body fur reduction, in a balance between such hindrances and safe infant transportation. Fur reduction is proposed to correlate with cooling mechanism in intra-species physical fights. The triggering of a feed-back mechanism connecting reduction of body fur to canine reduction would be responsible for a passage from threat displays to actual physical fights. The proposed scenario for such changes is the transition from uni-male to multi-male social structures among Hominoidea. The implications of the approach adopted are discussed.

##### Comments

The paper is based on the discovery of Lucy (*A. afarensis*), proving that bipedality was established 4 MYA, at the forest fringe, under a vegetable diet. The main reference (Berge et al., 1984) proposed that Lucy already had obstetrical traumatism, with rotation and flexion of the neonate in a ventrally oriented pelvic outlet, even with a small neonate fetal skull size. I added a further argument: bipedality is also unfavorable to safe pregnancy since it might favor abortion accidents, and presents problems of locomotion in the last months of pregnancy. Maternal and child mortality at birth ought to have been a major problem, and this will be discussed in this paper.

My original proposal evolved in two connected arguments:

– *The reproductive success was proposed to be proportional to the total infant survival probability, given by the product of two factors: Probability of survival of mother and fetus from conception to birth (obstetrical factor So) and probability of neonate survival during infancy (pediatric factor Sp).*
– *Reduction of body fur in hominids is the more natural candidate for the parallel process inducing bipedality, with a strong selective pressure for carrying infants on the arms of their mothers.*

A simple analysis, given in the paper, shows that body fur reduction brings bipedality as a NECESSARY CONSEQUENCE, before the condition of total nakedness is attained. A body fur reduction responsible for about 15% decrease in Sp, in relation to chimpanzee, would be enough to start to favor bipedalism.

Five basic references on physiology of hairs and skin support that reduction of body hairs comes before bipedalism: (de Beer, 1962; Schultz, 1968; Montagna, 1982, 1985; Ebling, 1985).

A main reference for my views on social structures of primates was published in Science (Eisenberg et al., 1972). Also interesting a short article on connections between canine reduction and the danger of canine wounds (Hutchinson, 1963), helping my proposal.

#### Paper 2—on Nakedness

The second paper (do Amaral, 1996) discusses the thermoregulatory advantages of hominid bipedalism combined with naked skin and larger body size. Published in Current Events, it does not have an abstract, it has 26 references and 6 Tables with numerical results of calculations. I question results from a specific paper favoring bipedalism after nakedness (Wheeler, 1992), with calculations for haired *vs.* naked bipeds and quadrupeds, crossing the four alternatives. I have reproduced several published curves using a scanner and Microsoft Windows on a PC computer, and performed numerical integration, making detailed discussions. I focus arguments of an earlier paper (Newman, 1970), that concludes “the obvious time and place where progressive denudation would have been least disadvantageous is the ancient forest habitat.” A critique to Wheeller’s ideas suggested that the advantages of bipedalism had little or no adaptive significance to thermoregulation (Chaplin et al., 1994).

I choose here some phrases that synthesize my Paper 2:

*“Although it is widely accepted that naked skin facilitates dissipation of body heat, the circumstances favoring its evolution are quite unclear. The point made in this paper is that although Wheeler’s calculations demonstrate the thermoregulatory advantages of bipedalism over quadrupedalism and of increased body size in savanna environments, the results do not indicate that the initial step in the denudation process occurred in open hot environments, nor that bipedality preceded body-hair reduction.”*
*“Wheeler’s work did made the point that nakedness has thermoregulatory advantages regarding water consumption at T_200_* = *30°C. However, this is interpreted here as suggesting that nakedness evolved in a more forested environment, and possibly, before or together with bipedality, not after it.”*
*“A more dry forest, which is one of the forest types of tropical regions*……, *could be a possible candidate as a habitat for the emergence of earlier hominids.*

These conclusions were confirmed with discoveries of fossils in following years, and are integrated in the accepted views on HE today. The origin of the process of hair reduction remains, however, unknown and continues to be attributed to thermoregulatory requirements. Recent revivals of this problem should be mentioned, and will be discussed in this paper.

#### Paper 3—on Infant Carrying

In order to give support to my proposals I needed to have experimental results, but it was not trivial to get samples of hominoid hairs. I went to a congress in France, and took the occasion to contact Dr. Christine Berge (Museum National d’Histoire Naturelle, Paris, France), and she gave me 3 pieces of skin with hairs, making this work possible (Amaral, 2008).

This paper has free access from internet. It has 50 references, 5 figures, 2 Tables. The relevance and pertinence of this original analysis for Human Evolution will be focused in the following items of Results. Here I make some general remarks on hairs as natural fibers.

Mechanical studies of animal fibers have been extensively performed because of interest in textile production, mainly wool (Feughelman, 1997) as well as cosmetics for human head hair (Robbins, 1994). Animal hair is composed of three parts: an external thin cuticle, a thicker cortex with fibrous proteins, and a central porous medulla.

**FIGURE 1:**
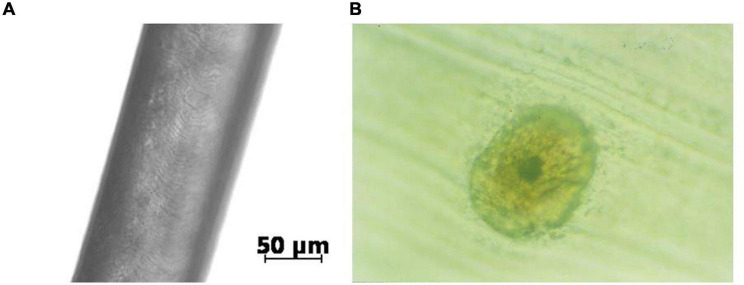
An example of ape hair (from orangutan) view in a microscope, Amaral (2008). The external appearance with the cuticle scale structure is shown in panel **(A)** and the cross-section inner structure in panel **(B)**.

Ape hair viewed under an optical microscope is similar in structure to wool and human head hair, as seen in Figure 1.

Mechanical properties of natural fibers are defined by the cortex of the fiber and are due to the molecular structure of keratin, which constitutes the cortex. Extensive work during decades evidenced the dependence of mechanical properties essentially on the cross-sectional area of the fiber, thus enabling basic research to be conducted on relatively few single fibers, eliminating the need for statistical methods on a large number of samples (Feughelman, 1997).

### The Problem of Infant Carrying

The following items give results from literature on infant carrying by primates, and also more details of the results obtained in my paper 3 (Amaral, 2008) in the context of biophysical analysis of the carrying problem.

### The Importance of Infant Carrying in Primates

All higher primates (except humans) carry their young clinging to their fur from birth. The correlation between infant carrying and the form of locomotion of adult primates is clear, but no detailed study had focused on the mechanics of the problem, until my detailed study from the point of view of biophysics of the carrying problem (Amaral, 2008), where references to infant carrying in primates can be found: (DeVore, 1965; Jolly, 1972; Tuttle and Watts, 1985).

Here some recent references are added, and the details necessary for an easy understanding by human scientists are given.

It is clear that safety in infant carrying imposes limits on the weight of infants, especially among Hominoids (our lineage), characterized by increasing body size. Accepted hominoid phylogeny places the branch to the Lesser Apes (gibbon) as the oldest, followed by the Great Apes, with the older branch to the arboreal orangutan and the branch originating terrestrial gorilla and chimpanzee coming later, together with biped hominins. Some discussion remains on the relative position of humans and the Apes (Ruvolo et al., 1994; Lockwood et al., 2004).

Among non-human primates, there is a change in the carrying pattern of infants by adults (mostly by the mother) as the infant grows. Newborns are carried clinging in close ventro-ventral contact, often with additional support from the mother. Change to infant support over the adult body (dorsal or lumbar clinging) occurs some months later (typically for infants heavier than 5 Kg) in all non-human higher primates and extends for years in apes. Besides all previous references, interesting results refer to evolution of infant carrying in primates (Ross, 2001) and co-evolution between infant carrying and grasping behavior (Peckre et al., 2016).

Examples of infant carrying by primates, to illustrate the comparison between monkeys (primates with tail) and Apes (primates without a tail), were given in (Amaral, 2014).

**FIGURE 2:**
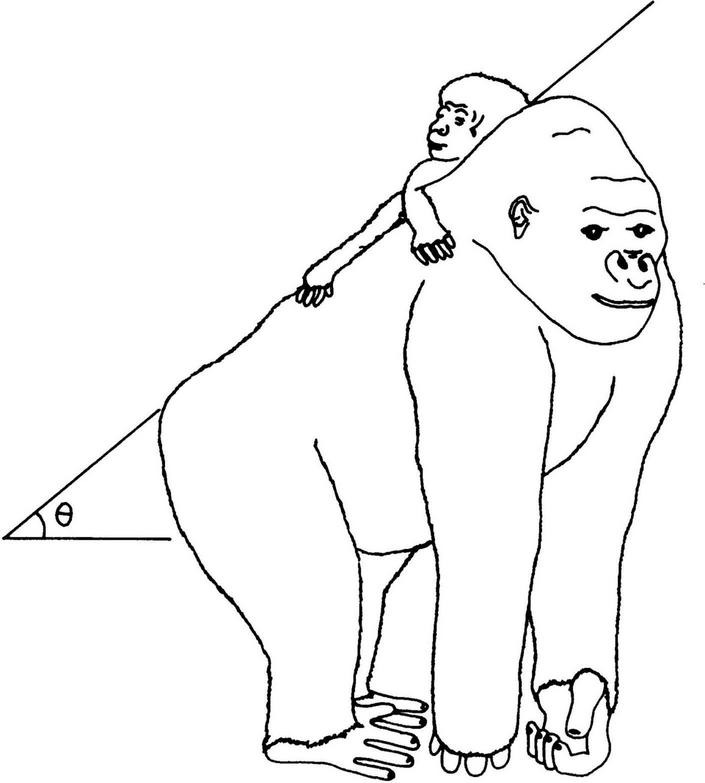
Drawing (done by João Carlos Terassi) of the simplified mechanical problem, with an angle of inclination, discussed in the text and also in Amaral (2008).

Monkeys carry the newborn clinging to the body hairs of adults in a ventral position for some months, until the infant is able to change to the mounting position. The tail of the infant is rolled up around the tail of the adult, usually the mother among African monkeys, helping the stability on movement (Figure 1A; Amaral, 2014). The Lesser Apes (gibbon) has a unique situation, with bipedal walk in tree branches and acrobatic movements of mothers with infants, easily found in internet photos, and also in a text posted by Sojourner (2011). The bigger arboreal Asian Orangutan (Great Ape) is suspended in a tree, but the mother holds the infant with one arm (Figure 2; Amaral, 2014). The larger terrestrial African Gorilla (Great Ape) mother is transporting the infant in the mounting position (Figure 1B; Amaral, 2014).

The sketch in Figure 2 is a simplification of the mechanical problem of the infant in an inclined plane, in the dorsal clinging to the gorilla mother (Amaral, 2008). Adult gorilla is much heavier than adult human, but their newborns have only half the weight of human babies. In the first 1 or 2 months, the infant gorilla is supported manually by its mother as she walks tripedally or bipedally.

**FIGURE 3:**
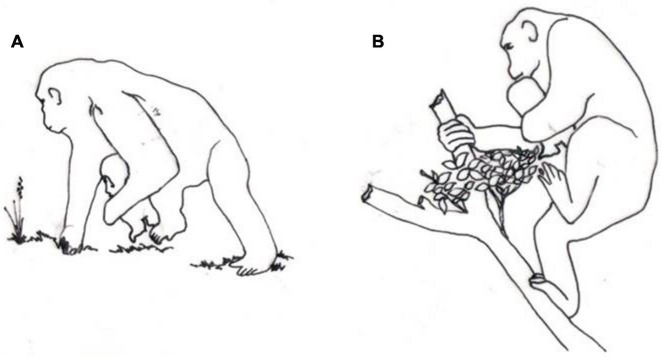
Drawings (done by João Carlos Terassi) inspired in photos (van Lawick-Goodall, 1967) of chimpanzee mother using tripedalism. **(A)** Using one hand to hold infant, walking in terrestrial substrate. **(B)** Using one hand to hold infant, walking in arboreal substrate.

As shown in Figures 3A,B, the same occurs for chimpanzee babies, unable to support their own weight by clinging prior to 2 months of age. Change from quadruped to triped or biped motion occurs systematically among all great apes when infant safety requires manual support. Slow and careful locomotion of female chimpanzees while carrying young infants has been reported, and two primary causes of mortality have been found among infant chimpanzees: inadequacy of the mother–infant bond and injuries caused by falling from the mother (van Lawick-Goodall, 1967).

It is clear that infant carrying is crucial and depends on not trivial behavior among African great apes.

#### Mechanics of the Carrying Problem

The mechanical analysis of infant carrying among Hominoids has been made along two directions (Amaral, 2008) with results shown also here.

##### Hair Tensile Properties

**FIGURE 4:**
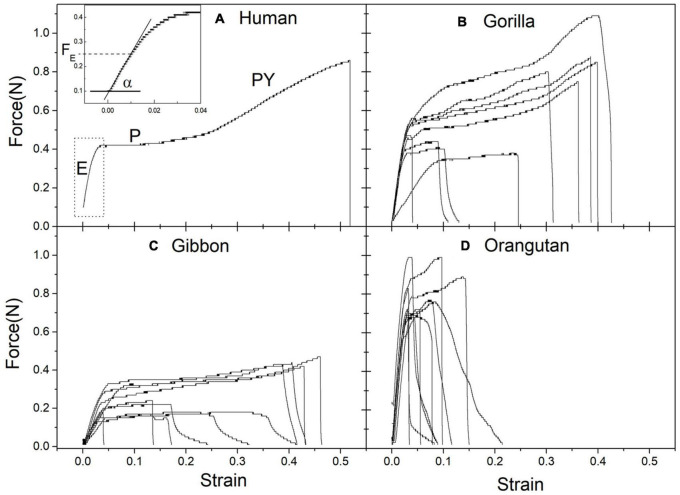
Experimentally obtained stress (Force) strain (relative deformation) curves for single hairs: **(A)** humans **(B)** Gorilla **(C)** Gibbon **(D)** Orangutan. Typical regions are seen: elastic (E), plastic (P) post yield (PY) and final break. In panel **(A)** the insert shows the linear region with coefficient α. See text for explanations.

The results to be discussed in detail came from three pieces of skin with hairs, each with about 200 cm^2^ area, obtained from 3 individual animals (one gibbon, one orangutan, and one gorilla—chimpanzee was not available), as already described (Amaral, 2008). The elasticity and the resistance capacity of the hairs are measured in stress/strain curves, one for each hair, as displayed in Figure 4.

Stress-strain curves for Ape hairs and human hairs for comparison: (Figure 4A) human, (Figure 4B) gorilla, (Figure 4C) gibbon and (Figure 4D) orangutan. The curves give the deformation (given as a fraction of the original elongation) due to the applied force (given in Newtons), until the breakage of the individual hair. Ten hairs of each individual have been measured. In Figure 4A, the region E corresponds to the linear region, P to the plastic plateau, PY to the “pos-yield” region, until the break of the hair. The insertion in Figure 4A is an amplification of the linear region, permitting to obtain the elastic force F_E_ and the linear coefficient α.

The curves display all the classical regions of animal hair. There is an initial elastic region, following a linear Hooke law, until about 1% deformation (elastic limit F_E_), followed by a plastic region, due to changes in the conformation of the keratin molecule, which is influenced by the amount of water, and a final break of the hair.

Curves are typical of all natural fibers, and in the human hair reveal the treatment given to it. The regression coefficient of the initial linear region α gives the Young modulus of elasticity Y, in units of pressure (GPa = 10^9^ N/m^2^), through the relation Y = α/A, where A is the area of the cross section of the hair, Ø its diameter, so that A = π (Ø/2)^2^. The elastic limit F_E_ is the limit that the hair can stand, in order to be able to support the pressure given by infant clinging to the hair. From each curve it is possible to measure the values of α and F_E_ (as shown in the insert in the figure), and also the values at the rupture point (force Fr and strain Sr). The force to rip out the hair from the skin has also been measured (20 hairs in each sample) and it is a little larger than the elastic limit.

It should be stressed the dependence between the hair diameter and the Young’s modulus of a particular animal species (Šimková et al., 2013).

It is clear from Figure 4 that the results are different for each hominoid species. Furthermore, the skin and hair from the different ape species are easily recognized by appearance and texture: Gibbon hairs are black, thin and smooth silky, those from orangutan are reddish, thicker and rough, while those of gorilla are black with intermediate thickness and texture.

The important point to emphasize is that properties of hairs are “species characteristic,” and therefore even a single individual is enough to obtain meaningful results, extensive to that species. In the original paper (Amaral, 2008) the conventional statistical analysis, used in biology, has been made, since hairs are a “natural biological sample.” Differences between species are statistically significant, in the comparison of each pair of species, and also in the Anova test for the 3 ape species. An extensive discussion on the implications of the results regarding safety of infant carrying along our evolutionary line was already published and will not be repeated here.

##### Mounting Position of Gorilla Infants

Let us analyze the dorsal clinging position, following paper 3 (Amaral, 2008). Figure 2 is a sketch of the mounting position in African apes, showing the angle θ of the inclined plane where the infant stands. In the sketch the angle θ coincides with the angle defined by the usual knuckle-walking position of African great apes, this just helps to capture the forces in action. The total infant weight, in the vertical direction, has two components, one in the inclined plane, favoring slipping, and one normal to the inclined plane, responsible for the friction force opposing slipping, which depends on the friction coefficient. In the absence of clinging, slipping on the body surface of contact starts for a critical value θ_c_ and the infant falls down. Both friction and clinging are thus essential to hold heavy infants in the mounting position.

It should be stressed that this is a necessary condition for infant survival and therefore also for species continuation. It is a very robust requirement of basic mechanics for equilibrium. The ape hair–hair friction coefficient μ must be known to analyze the actual ape situation, and it was actually measured and discussed. This simplified mechanical analysis is able to explain the observed angle θ presented by Gorillas (estimated in about 26°) (Amaral, 2008).

A reference to my paper (DeSilva, 2011) mentioned *“the elimination of dorsal riding as an option for infant hominids,”* in the context of bipedality. deSilva work proposed a shift toward birthing relatively large infants early in human evolution, analyzing the infant/mother mass ratios in Australopithecus females.

## Discussion: Hypothesis and Theories

There exist bias strongly acting against acceptance of new ideas coming from “outsiders” (my case) and their integration in the *status quo* of the main stream in “academic knowledge.” This is quite obvious regarding Human Evolution, strongly connected with religion and social habits and costumes. But what became clear to me now is that a main bias seems to exist also between Exact Sciences and Biological Sciences, and it has to do with a belief on the power of Mathematics to solve any problem, what is of course not true. Any theory relies on initial assumptions, taken for granted as intuitive and without proofs. This forms a very rigid body of ideological beliefs, not open to real debate.

This discussion will first enter on rational arguments, and only later will attempt to overcome such bias.

### Infant Carrying and Human Birth

Let us discuss proposals that go also in the direction of “infant carrying” leading to bipedalism, with connections with the obstetrical problem.

Iwamoto (1985) recalled examples of facultative bipedalism among monkeys and speculated that the “decisive factor (for habitual bipedalism) may have been some everyday necessity to carry something in both hands.” After criticizing proposals in which the “something” is food, Iwamoto suggested that the “something” could be their helpless babies. However, this proposal has been dismissed under the hypothesis that babies became helpless only with increase of brain in Homo.

The work by Berge et al. (1984) showed that babies were helpless before increase of the brain, but her work focused in “obstetrics,” did not enter really on the connection to bipedalism. The same is valid for all later studies on parturition. The very detailed paper by Rosenberg (1992) enters on birth in non-human primates and on the evolution of modern childbirth with its overwhelming importance in human evolution. But she also did not come to the point of proposing a direct connection to the evolution of bipedalism.

The correlations between changes in the pelvis, occurring in the earlier stages of evolution of erect posture and the obstetrical mechanism of females had been pointed out long time ago (Robinson, 1972). A very interesting discussion (Robinson et al., 1972) made on the difference in bipedal efficiency of pongids and hominids, suggested that “*the evolution of erect bipedality appears to have occurred in two phases, the: first of which was primarily concerned with re-positioning the center of gravity to reduce the energy cost of frequent use of bipedality, and the: second concerned the development of a speed-oriented striding and running mechanism.”*

Such two phase process is now very well established, since bipedality evolved much before the erect position.

At the same time that my third paper was available in the internet (Amaral, 2008) the paper on fetal load and the evolution of lumbar lordosis in bipedal hominins was also published (Whitcome et al., 2007): *“human females have evolved a derived curvature and reinforcement of the lumbar vertebrae to compensate for this bipedal obstetric load. Similarly dimorphic morphologies in fossil vertebrae of Australopithecus suggest that this adaptation to fetal load preceded the evolution of Homo.”*

However, the connections between these findings on lordosis and my proposal on infant carrying were clear only for me:

– The problem is evident only in the three last months of pregnancy. But of course infant carrying would represent a much bigger and long lasting effect.

From this perception, I went in two different directions. In my university I proposed collaboration with a physicist working at the Faculty of Sports, in a Laboratory of Biomechanics. My idea was to study the locomotion of mothers carrying their babies and the effects on their movement, focusing on the pelvic and spinal posture and the displacement of the body’s center of gravity.

The second direction came from my direct contact with Karen Rosenberg (United States), who proposed a Symposium on Infant Carrying in Human Evolution at the 78th annual Meeting of the American Association of Physical Anthropologists, Chicago, April 2009. The 15 invited persons for oral presentations included me and Katherine Whitcome. So I attended the meeting, with an abstract published (Amaral, 2009). In that meeting I met an American anthropologist and started collaboration with him. In the following year I attended also the 79^th^ meeting, in Albuquerque, New Mexico, April 2010, also with a published abstract (Williams et al., 2010).

But it became clear to me that my original proposal, focusing the correlation with reduction of body hairs, was not evolving internationally. The collaborations I could start extended over years, in parallel with my regular activities in Physics, leading to two articles in HE:

– Sex differences in forearm proportions (Williams et al., 2015).
– Effects of transporting an infant on the posture of women (Junqueira et al., 2015).

I decided then to turn to divulgation of my proposal in my ambient, with two papers in Portuguese (Amaral, 2014; do Amaral, 2015). I also started to present seminars on HE, when invited by colleagues who knew my activities in Physics. I gave such seminars in the Institutes of Chemistry, of Pharmacy, in Medical Schools, even in the Biology. The Institute of Theoretical Physics invited me only within the Divulgation seminars, not in the official ones, and in my own Institute of Physics also.

I noticed then that the audience could be very receptive to my proposal, when the scientists did not have compromises with the mainstream in HE. Moreover, women in general accepted it as “obvious,” while men in general “laughed” as hearing a joke! The “structural chauvinism” became very clear. I then entered in the problems related to History of Science and the terrible difficulties due to the mainstream blockage, of social nature.

When the pandemic arrived a very long time at home started, working alone in my computer. But online events started to exist all over the world, opening new opportunities.

### Looking Again at Data From Hominoid Hairs

My data on Hominoid Hairs, shown and discussed in previous items could be published (Amaral, 2008) only after I did all the conventional statistics used in Biology, which are not used in Physics. I started then to think seriously on the differences between statistics used in Physics and statistics used in Biology and Human Sciences. They are based on different principles, and I started to study the problem from a historical perspective, already in the 2 years before the pandemic. Then in 2021 appeared the opportunity to attend an online meeting on Statistics, I presented an oral paper, published in the Proceedings (do Amaral, 2021), on Statistical Analysis of Measurements in Exact and Inexact Sciences, which are in fact an Open Problem until today, and I copy here its Abstract:

#### Abstract

Differences between statistical analysis of measurements in exact and inexact sciences are the focus of this work. The early and independent beginning of Probability and Statistics had a theoretical synthesis, with an initial development based in Physics and Astronomy. This lead to Error Theory, used in Statistics of Measurements in Exact sciences, with defined criteria of validity. This direction of Mathematical Physics resulted in the progresses and achievements in Classical Physics, and also on established ways of treating measurements of physical properties. It is discussed that Exact Sciences treat only Inanimate Matter, and things that can be defined and measured, in terms of only seven fundamental physical quantities, with the definition of the International System of Units (SI). On the other hand a direction of Mathematical Statistics emerged later on, based on “Sampling,” to study properties of a population, with criteria of significance, within validity intervals, which depend on the size and characteristics of the studied sample, and on the inferences to be made in the research. These are two very different approaches, but both use probability density functions related to hypothesis about data. The modern inferential sampling statistics can be applied to all practical problems, in particular in Biology and Humanities, where there are “models,” but not Theories as in Physics. The word “theory” is many times used in a mistaken way. Life and Human Sciences use this modern type of Statistics. This paper discusses a particular case, in which the same ensemble of experimental results in samples of biological origin (hairs from hominoids) can be analyzed with the two different statistical approaches, in a proposal for Human Evolution, and the conditions for inference of accurate conclusions are discussed. A philosophical discussion between subjective and objective criteria of the researcher is made, and also of the concept of knowledge.

#### Reanalysis of Data

Here previously obtained data on hairs (Amaral, 2008) is re-analyzed with presentation of Table 1, constructed from the original data, allowing a more simplified discussion, as presented in that recent conference (do Amaral, 2021).

**TABLE 1:** Thickness of the skin and length of hair (estimated) average values of the measured values of the hair diameter ∅, of the linear regression coefficient α, of the elastic limit F_E_, and from Young modulus Y.

	Gorilla	Gibbon	Orangutan	Human
Skin thickness (mm)	∼1.5	∼0.6	∼2.3	
Hair Length (cm)	∼6	∼4	∼10	
∅ (μm)	66 ± 2 (21%)	52 ± 2 (19%)	120 ± 4 (24%)	60–80 ethnic determined
α (N)	17 ± 2 (25%)	6.0 ± 0.6 (33%)	32 ± 1 (13%)	
F_E_ (N)	0.38 ± 0.03 (30%)	0.18 ± 0.02 (36%)	0.49 ± 0.02 (12%)	0.28 ± 0.02 (29%)
Y (GPa)	5.0 ± 0.6 (42%)	2.8 ± 0.3 (43%)	2.8 ± 0.2 (36%)	2.9 ± 0.2 (33%)
Weight Adult (Kg)	150–250	7	50–90	
Hair Density hairs/cm^2^	100	1,000	∼200	

*Constructed from original data (Amaral, 2008; do Amaral, 2021). Averages show the standard deviation of the mean, in parentheses the coefficient CV = 100 × standard deviation/average value. Final lines show the weight of the adult animals and the density of hairs of each species.*

The symbols for the variables in the first column were given, when explaining the stress/strain curves shown in Figure 4. The legend on Table 1 explains the values shown in this Table.

I stress here what is necessary to a better understanding of the issue. The more important variables obtained from the stress/strain curves are:

– elastic limit F_E_ (N), which can also be given in units of gf (gram-force ∼ 0.01 Newton), a direct measure of the weight the single hair can stand without a permanent deformation.
– regression coefficient α of the initial linear region, which together with hair diameter ∅ (μm) gives the Young modulus of elasticity Y.

The values given in the columns show the average values obtained from the 10 measured curves, with ± the “error” (standard deviation of the mean). In parentheses is the variation coefficient, related to the broadness of the distribution.

This table allows an interesting comparison with the statistical analysis required in biology, in the published paper (Amaral, 2008).

The sampling statistics used in biology is certainly very helpful, but many times the amount of available samples does not allow a decision only on basis of a “large amount of data.” In this case there are solid arguments in favor of accepting the approach used in physical sciences, when the study is made on “known samples.” It corresponds to adding “extra known information” to the available data.

That is indeed the case for “hairs”:

– it is well established that analysis of hair properties is used in legal forensic science to determine the animal species, and this is valid also for human hairs, as seen in a book (Robertson, 1999), with a book review (Tridico, 2001) and also on a review published by FBI (Oien, 2009).
– it is well established that measurements can be performed in relatively few single fibers (Feughelman, 1997).

In physical sciences the research is usually made in one sample of a known material, with repetition of measurements of a physical property. So that 10 independent measurements of hairs from one individual may represent a sufficient ensemble, since it is known for sure that the individual is from a specific ape species. Extra information enters the statistical ensemble of data, since it is known and accepted that the 3 individuals represent each one a separate species.

In physics it is not usual the use of statistical analysis in terms of the parameter “*t* of student” as function of the degree of freedom and confidence level. Analysis uses simply Gaussian function, when applicable, since the probability of the distance of a measurement to the average value depends on the standard deviation σ, in a known way:

– 68.2, 95.4, and 99.7% probability for the measure to be within, respectively, 1σ, 2σ, and 3σ from the average value.

Comparing Table 1 above with the previous sampling statistics (Table 2 of Amaral, 2008) it is possible to say that the “thumb rule” of a physicist holds:

– if two independent average values differ by more than 3 × “error” they are most probably NOT measuring the same thing.

The same inference can be made here, since there is enough separation between the values of all measured parameters for the different species.

This discussion needs some more information regarding the two last lines of Table 1, showing the weight of the adult animals and the density of hairs of each species.

### Hair Density in Primates

Reference to the work of Shultz on hominoid primates was mentioned already in my first paper (Schultz, 1968). In my third paper the question of density of hairs in non-human primates was discussed in detail, with reference to the extensive work of Schultz showing that hair density values on the back of primates vary, from over 1,000 hairs/cm^2^ for smaller species (monkeys and gibbons), down to about 100 hairs/cm^2^ for great apes (Schultz, 1931).

This very large amount of data was analyzed 50 years later (Schwartz and Rosenblum, 1981) resulting a scaling law with a negative allometry of relative hair density (rhd = hair density/total surface area), as seen in their paper. There is a decreasing scale law among primates, relating the log of the relative density of hairs rhd with the log of total surface area (cm^2^). And it is clear that “*chimpanzees apparently have a lower rhd than would be expected for their body volume.”*

These results were integrated in the discussion made in my paper 3. Among Hominoidea, the small and light Gibbon is very hairy, while the big and heavy Gorilla has a density of hairs 10 times smaller. This represents a clear problem in the usual primate infant transportation, clinging to the mother’s hairs.

In order to make now a more complete discussion on the problem of infant carrying the weight of the adult animals and the density of hairs of each species are added in the last lines of Table 1.

Moreover, a recent communication with a detailed comparison with mammals (Sandel, 2013) confirmed a significant negative correlation between hair density and body mass. Results indicate that all primates, and chimpanzees in particular, are relatively hairless compared to other mammals. Humans are covered with small fine vellus hairs, not counted as terminal hairs. The meaning of this clear scaling law is not yet completely understood. But its effect on the problem of infant carrying is very clear. There is a defined increase in weight along phylogenetic evolution: gibbon—orangutan—gorilla, infants became heavier, but the density of hair decreases, and safe carrying of heavy infants becomes THE problem to be solved.

Taken into account all the available information, it is possible to infer a pattern about evolution, from the obtained results of hairs of the three species. The increase in size and weight in the Asian line occurs with the same young modulus Y, with large increase in hair diameter ∅ (μm) and elastic limit F_E_ (gf) from Gibbon to Orangutan. The African direction, with the much heavier Gorilla, required a larger value of the Young modulus Y, allowing for not so large increase in hair diameter ∅ (μm) and elastic limit F_E_ (gf). The case of chimpanzee will be discussed later on.

**FIGURE 5:**
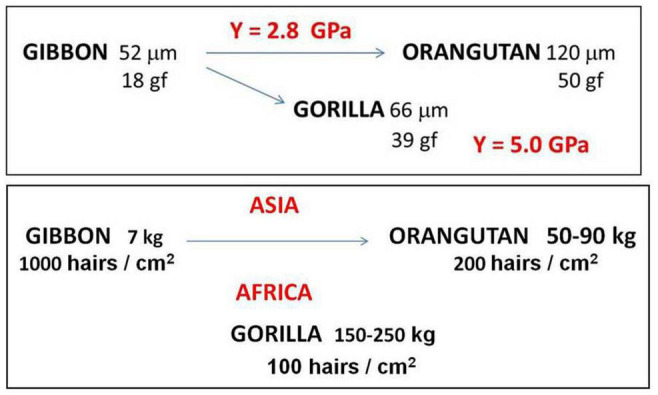
Sketch of evolution among Hominoidea: upper focusing the Young modulus, down the species location. See text for complete explanation.

Figure 5 display a double sketch of the evolution in terms of the important parameters obtained from mechanical analysis of hair properties of the three hominoid species: the hair diameter (in μm), the elastic limit (in gf) and the Young modulus of elasticity (in GPa) in the upper sketch, and the lower one with the weight (in kg), the hair density (in hairs/cm^2^) and the geographic location.

The same Young modulus was obtained for gibbon and orangutan, along arboreal evolution in Asia, while a much larger Young modulus was obtained for terrestrial largest Gorilla. So that Gorilla hair can stand much more weight than orangutan hair. Of course all this is just an “indication” and much more studies are necessary.

**FIGURE 6:**
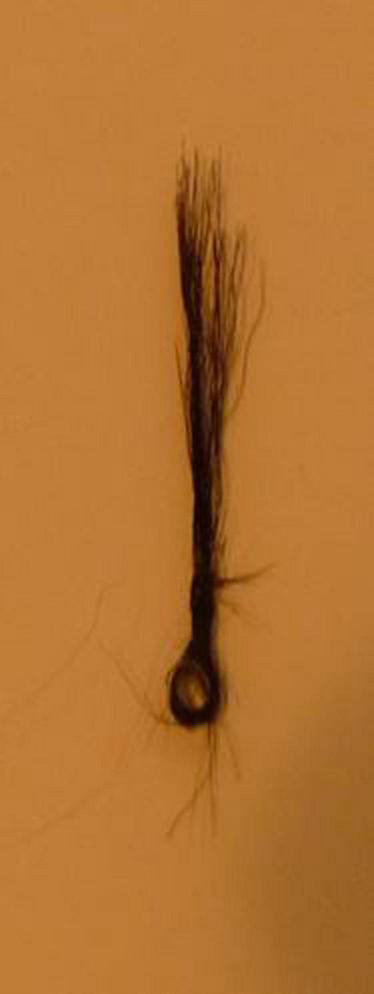
Result of experiment to test the weight limit for the bunch of hairs. See text for complete explanation.

The discussion in Amaral (2008) considered also that the bunch of hairs available for clinging depends also on hair length and infant hand size, so that bunches of about 100 hairs are necessary to carry infants weighing a few kilogram-force. To simulate the real load effect in the actual clinging situation a bunch of hairs on about 1 cm^2^ (± 10%) of gorilla skin under load was observed, still on the skin. The bunch of hairs broke, detaching from the skin, and Figure 6 shows the bunch after detachment from the skin. This occurred under a static load of 7 kg, compatible with the values of force at rupture (Fr) from the stress/strain curves of Figure 4.

– An upper limit of 1 kgf/cm^2^ for clinging without problems on the hominoid skin may be deduced from this simple experiment.

Humans cannot be placed in the primate scale law regarding fur, the denudation process is unexplained, but chimpanzee already deviates, having the same density of hairs as gorillas, but being much smaller, with weight about 1/3 of the gorilla.

Conclusion: Hair physical properties might have evolved correlated to the pressure of safe infant carrying, considering the change from arboreal to terrestrial locomotion.

It should be stressed that dermatology encompass both skin and hairs but they are physically different. Human skin, with all its unique properties, will be mentioned later on.

### Ideological Bias Prevent Understanding of Human Evolution

As mentioned in the beginning of this Discussion, after rational discussion, it is now necessary to enter on the problem of ideological bias. First point is related to the tripedal locomotion of gorillas and chimpanzees, discussed in the text and shown in Figure 3.

The small gibbon has ten times more hair density than the large gorilla, and about 30 times less weight than the Gorilla. Gibbon infants are safely carried, clinging to the mother’s hairs, while the Gorilla newborn requires that the mother hold it with one hand, walking tripedally for some months, until the infant is able to adopt the mounting position.

It seems clear that tripedal walk, occurring naturally among African Apes carrying their newborns, unable to support themselves by ventral clinging, is the natural candidate leading to evolution of a bipedal locomotion.

The idea of tripedal walk as an intermediate stage to bipedalism was in fact theoretically proposed (Kelly, 2001), based on the fact that human anatomy is more asymmetric than in the Great Apes, but with the hypothesis that it came from the need to through stones for defense. In other words, rational arguments are mixed with a focus only on males!

I start now to make new proposals, not published yet, as HYPOTHESIS:

– Reduction of body hairs extended the length in time of tripedal locomotion, initially of some months, to the point that it became more effective to adopt bipedalism.

This hypothesis joins the tripedal theory worked by Kelly to the theory I formulated in my paper 1, considering the reproductive success to be proportional to the total infant survival probability, given by the product of two factors, as proposed in do Amaral (1989) and mentioned previously here.

In previous items the difficulies between methodology of Exact Sciences and Biological Sciences have been rationally discussed in the context of results on mechanical properties of Hairs. But it is necessary now to face the main ideological problem:

– There has been always a very strong resistance in the academy to the idea that bipedalism could be connected to a “female problem,” that means, that it was not a “male acquisition.”

Models are in fact developed from the point of view of the adult male individual. Here it will be instead focused the female perspective, including the connection with infants, which is the most important for reproduction and survival. With such perspective this discussion returns to Sexual Selection.

### Sexual Selection Among Hominoids

There is a pattern in the evolution of hominoid socio-sexual structures, of not easy explanation, as discussed in my first paper: Gibbons are small and mainly monogamous, larger Orangutans are solitary and heaviest Gorillas have harém, but none of them accept coexistence with other adult male. Growth in size, together with large sexual dimorphism, indicates sexual selection among Orangutan and Gorilla. Chimpanzees have a fission–fusion promiscuous system, males are genetically related in their groups, while Humans are flexible. The whole process of initial separation of humans from the ape lineage is unknown.

From the global overview on the problem, my proposal published more than 30 years ago was:

*“The proposed scenario for such changes is the transition from uni-male to multi-male social structures among Hominoidea”.*

**FIGURE 7:**
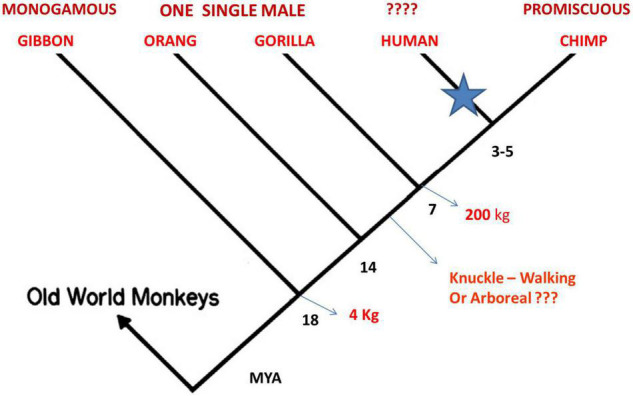
Accepted phylogenic evolution, based on DNA analysis (Lockwood et al., 2004) placing Homo between Gorilla and Chimpanzees. Extra information pertinent to this discussion is shown in red. See text for the proposed explanation for split of the hominin lineage.

This discussion intends to show that the scenario proposed 30 years ago remains compatible with all knowledge accumulated with the extensive theoretical and experimental work done on primates in the last decades. Figure 7 shows a sketch of phylogenetic evolution of Hominoids, including Lesser Apes, Great Apes and Humans. Most primate groups of Old World monkeys are organized among related females while among Great Apes genetic relations are between males.

Let us discuss the global picture that emerges from comparison of some recent reviews with very detailed results on primates: (Plavcan, 2001; Dixson, 2012; Fleagle, 2013). What becomes clear is that all primates must be considered, in comparison with humans, in order to detect what is common and what is specific. There is not total agreement on the exact phylogenetic tree, there is preference for placing Humans separated from chimpanzees, stressing similarities between humans and all primates, since the correlation between genes and behavior is not clear and some parts of the human genome are more similar to that of gorillas than chimpanzees. The total percentage of equal genes says nothing about specific differences. More agreement exists on the definition of “species.”

These recent reviews agree that the socio-sexual behavior of primates is very flexible, with space to work indefinitely in all the details deviating from the averages in humans as well as in all primate species, but my aim is to understand the basic trends, in order to achieve a general view able to overpass the existing bias. The most obvious secondary sex differences among primates are body mass dimorphism, and canine tooth size dimorphism, but pelage dimorphism is also present in several species and can be quite impressive. In fact the genitalia and secondary sexual traits evolve in both sexes for effective reproduction.

It is worth stressing some specific outcomes from these recent reviews:

– Plavcan stresses that sexual dimorphism is the product of changes in both male and female traits, and each sex compete agonistically in the context of coalitions (Plavcan, 2001).
– Dixson’s second edition book emphasize that there is not a rigid dichotomy between the “sexual” and “socio−sexual” functions of behavior and discusses the effects of hormones in the sexual behavior (Dixson, 2012).
– Feagle’s third edition book stresses that all great apes build nests for sleeping and resting, but share few unifying features in their social behavior, and describe non-trivial male–female sexual encounters among Orangutan (Fleagle, 2013).

### Proposal for the Split Toward the Human Lineage

The term hominin refers to species on our branch of the hominoid tree after the split with the chimpanzee and bonobo line, including all of the extinct species and evolutionary side branches (Wood and Lonergan, 2008).

The proposal presented here is that the hominin lineage evolved from a critical transition from unimale to multimale social groups, with intense tension between sexes and rather complex intra-sexual and inter-sexual interactions. Intense socio—sexual behavior might correlate with bursts of internal heat, requiring less hair density for body cooling. Thus an initial reduction of body hairs occurred in the ancestors of both chimpanzees and naked bipeds, related to intra species aggression.

A clear result from fossil evidence is the very early reduction of canine size in the hominin lineage, correlated with changes in the socio-sexual interactions. Reduction of canine size does not correlate with monogamy, since monogamous gibbons have large canines in both males and females, and also because fossils indicate large sexual dimorphism in early hominins. Here it is recalled the idea (Hutchinson, 1963) that canines are a too dangerous weapon when successive generations started to coexist within a group, particularly if an early process of fur reduction increased the danger of canine wounds. The inevitability of the process was stressed by Hutchinson.

Hypothesis: reduction of body hairs came together with canine reduction, correlated with passage from threat displays to actual socio-sexual physical fights, with body contact. Such intra species physical fights are much more frequent among humans than among other mammals.

Reduction of body hairs together with bipedalism means that females must carry infants on their arms, have no free hands to get food, and need help to survive. The more natural solution is the formation of female kin groups, with males floating around the periphery, and mating occurring at special times. This is coherent with considering the split toward the hominin lineage within the whole primate behavior. It is from this perspective that the basic question of the naked skin is discussed in the next item.

### Thermoregulation and the Naked Skin

The still unsolved question connecting naked skin with thermoregulation must now be faced. More recently the modeling of heat balance was extended to consider a running hominin, with the conclusion that *“only when hair loss and sweating ability reach near-modern human levels could hominins have been active in the heat of the day in hot, open environments”* (Ruxton and Wilkinson, 2011a,b). This can be conciliated with a very early fur reduction, considering that earlier hominins lived still in a forest ambient.

The model was again retaken, considering the altitude at which australopiths actually lived (David-Barrett and Dunbar, 2016). This last argument trying to place hair reduction after bipedalism mentions: *“cool night time temperatures would have made it impossible for substantial hair loss to have evolved in species occupying the sites where Australopiths appear to have lived in the absence of cultural* (e.g., *shelter, clothing) or other behavioral (nesting, group sleeping) developments”* (David-Barrett and Dunbar, 2016). In order to keep a very early process of hair reduction, the solution is indeed to focus on the possibility of behavioral nesting and group sleeping associated with a primitive shelter. Two arguments can be invoked now:

– Chimpanzees, and large bodied hominoids, construct every day their nests inclusive -with thermoregulatory capacity (Samson and Hunt, 2014).
– Along the last decade the concept of social thermoregulation among humans evolved in the psychological literature, with a defined theory (IJzerman et al., 2015).

This discussion turns now to the physiology of animals (Davenport, 1992; Schmidt-Nielsen, 1997) and humans (Bruck, 1989) giving support to arguments on biological thermoregulation as a physiological dynamical process. The practically constant basal metabolic rate (BMR), the minimal rate of energy expenditure per unit time by endothermic animals at rest, is very well known. The thermoneutral zone (TNZ) is defined as the range of ambient temperatures where the body can maintain its core temperature. The typically small tropical thermoneutral zone (TNZ), between 27°C and 30°C, represents a very small range of temperature tolerance in both directions, and the problem of altitudes in the tropics means that night temperatures of the order of 10°C must be considered.

Non-shivering thermogenesis (NST) is the increase of individual basal metabolism below the minimum critical temperature of the TNZ and participates in the chemical thermoregulation of mammals, especially neonates, connected to the metabolism of the brown adipose tissue (BAT). Extensive studies on tolerance to coldness show that NST can be induced by adaptation to cold and its molecular basis was found (Lowell and Spiegelman, 2000). A recent study has shown the shift of TNZ in a mamal (hamster) acclimated to different temperatures: cold (5°C), warm (21°C) and hot temperatures (31°C) (Zhao et al., 2014). A naked human shows increased heat production as environmental temperatures falls, and Davenport (1992) gives at 10°C a factor two in heat production in relation to TNZ. The conductance responsible for the blood flow from the core to the skin in humans can change by a factor 4 to 7 (Bruck, 1989), depending on the rate of blood change, and the thickness of the body shell and of the subcutaneous fat layer.

The real question to be discussed is how already hairless Australopithecus could solve the problem of coldness in the altitudes where they seem to have lived. Naked Australopithecus could be biologically well adapted to the tropical altitudes by a combination of NST (with a factor 2–3 over BMR), and primitive shelters made of natural materials, where a single naked adult individual may survive. However, for females with infants a real problem exists. A naked neonate has a high NST but a surface/body ratio ∼3 times higher than that of an adult and requires high ambient temperatures for survival (Bruck, 1989).

– The solution here proposed is that females with infants must huddle together for thermal cooperation.

Biological adaptations to coldness in many animal species include the cold-induced huddling, i.e., individuals together to regulate energy saving processes. This happens from penguins living in arctic conditions (Ancel et al., 1997; Gilbert et al., 2008), to penguins and birds in different l altitudes (Black et al., 2016) and also for small mammals, as studied in detail with rat pups (Alberts, 1978) and mouse development (Harshaw and Alberts, 2012; Harshaw et al., 2014) with very defined experimental results. The situation in a huddle corresponds to N bodies with individually negative heat flows, but the huddle body as a whole may have positive heat flow, as can be seen in thermographs of real biological huddles [for instance Figure 1 in Harshaw et al. (2014)].

The body surfaces of individuals in direct contact do not contribute to the huddle surface. For *N* = 2 the individuals are half within the huddle and half at its surface. For *N* = 3 the individual in the middle is really protected inside the huddle. These minimal huddles are compatible with the estimated efficiency of primitive shelters. The shelter efficiency defines the number N of individuals huddling, and only in good shelter and insulation conditions individuals may be independent.

It is possible now to discuss a very peculiar characteristic of the human skin, the clear sexual dimorphism regarding distribution of hair and also fat in the bodies of males and females. In order to arrive to a solution, it is necessary to discuss the thermoregulatory difference between a furred and a naked skin.

### Thermoregulation of Furred and Naked Skin

In furred animals the fur acts as an insulator, and the surface temperature of the fur can be equal to ambient temperature, depending on fur density and thickness, by trapping air, which is a good insulator. There is therefore no difference between surface and ambient temperature, with minimal heat losses to the ambient. The fur insulation value and the variation of metabolic rate with temperature define the adaptability of the species, in a very large interval of ambient temperatures (figures 7.7–7.9 in Schmidt-Nielsen, 1997). In the case of the human skin there is an insulating fat layer below the skin surface, and the heat balance is controlled by the flux of blood. The fat insulation can be bypassed for heat dissipation (figure 7.14 in Schmidt-Nielsen, 1997), and this is not possible in a furred animal, which uses other processes for change in body conductivity for heat balance. Clearly the human fat insulation evolved in the context of skin-air contact, not in skin-water contact, since the conductivity of water is 25 times higher than the air conductivity (Toner and McArdle, 1996), and the fat layer of normal humans is not so thick as the blubber of water mammals, neither does it exist in the whole body surface as it does in marine mammals.

Thermoregulation of human naked skin is thus defined by the flexibility of blood bypassing the subcutaneous insulating fat layer, allowing both insulation and refrigeration, in order to cope with hot and cold daily variations.

The crucial point to be stressed is that effective skin contact for the huddle solution is attained by naked skin in both mothers and infants. A recent study examined gender difference in human response to temperature step changes and results indicate that females differ from males in human response to sudden temperature changes from the perspectives of psychology, physiology and biomarkers (Xiong et al., 2015). Females are more prone to show thermal dissatisfaction to cool environments while males are more likely to feel thermal discomfort in warm environments. This result and similar ones performed recently give support to the proposal made here of an original huddle with only females with their children. This explains that loss of fur evolved together with the fat layer below the surface skin in females, and neonates.

**FIGURE 8:**
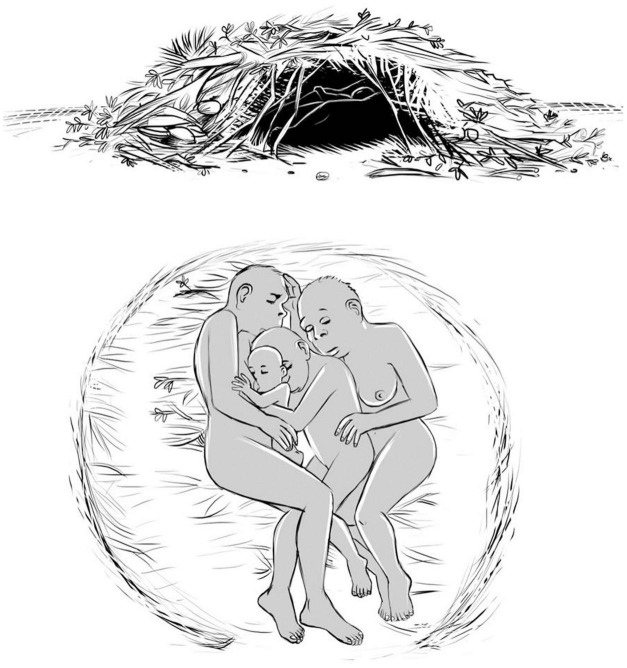
Sketch of the possible solution for earlier naked hominins: huddle with female family for thermoregulation in a primitive shelter of natural materials (done by Carlinhos Muller, following my original idea, see details in the text).

A sketch of this proposal is shown in Figure 8. Neonates and infants must be protected in the microclimate inside the huddle, and the minimal solution for hairless Australopithecus survival consists of few females, related by kinship, embracing and protecting infants in a primitive shelter. The infant bodies are smaller than the bodies of the adult females, so that they are in close contact with the female cores at ∼37°C. The infant bodies, with a high NST, have high temperatures, so that the female bodies receive also thermal protection.

This *biological* solution is in fact expected from primate social behavior, since all Great Apes live in small groups, but with different strategies in the two sexes.

#### Hypothesis for a Theory

– It is already known that Bipedalism evolved in two steps, the first one in australopithecines, the second one in Homo Erectus.
– The proposal made here is that reduction of hairs evolved also in two steps. The first one in Australopithecines, with nesting and group sleeping associated with a primitive shelter and close physical contact between few females, related by kinship, embracing and protecting infants in a primitive shelter. The second one in Homo Erectus, when conditions for walking and even running with locomotive efficiency, and sweating rates to be active in the heat of the day in hot, open environments.

### Model for Biological Evolution of Earliest Hominin Social Structures

The chimpanzee/bonobo lines arrived to some sort of socio-sexual equilibrium, stabilizing the reduction of body hairs, and keeping large canines. But the lineage of naked bipeds did not reach easily such stabilization, embarking in the direction of further reduction of body hairs, small canines, bipedalism, and separation of female and male sociality under specific sex defined behaviors.

The proposal made here is that naked Australopithecus evolved with different social structures for females and for males. Relations between sexes were possibly occasional and violent.

In female families, the naked females developed strong bonds with their naked infants, with permanent physical body contact, care and attachment, and kinship cooperation in female sociality. Breasts have a clear function for mother-infant contact, for nutrition and increase of warm skin contact. The basic biological changes in both sexes are directly related to efficient reproduction, the attractiveness comes as a consequence. Hominin females are more selective because they are extremely “K-selected,” in the modern concept of life-history evolution (Reznick et al., 2002) with large and long maternal investment on a single newborn.

A rite of passage from infancy to adulthood must have been the first basic rule of such groups, deeply imprinted in the psyche of naked Australopithecus, and biologically determined by hormone release, not by consciousness or intellectual capacity.

– The male infant changed pitch of voice with growth, and this defined the exit from the original female family to become a young adult male, either solitary or in bands of males.

These biological changes probably defined the structuring rites of passage already in naked Australopithecus, and subsist in all human primitive groups even today (van Gennep, 1909). A revision of anthropology and social sciences is in order, as done by Szakolczai and Thomassen on their book, where the first chapter is “Arnold van Gennep—Liminal Rites and the Rhythms of Life” (Szakolczai and Thomassen, 2019).

An example of study on evolutionary psychology discussing intrinsic versus extrinsic motivations in humans is given in a recent paper (Varella, 2021) on artistic motivation using a large decades long database of university applications together with career-choice reasons.

Considering all that is known about primate and human behavior, it can be argued that only in Homo males started to help the transport of older infants in their shoulders, and groups of males together with females could be formed.

## Darwin’s Legacy and Future Prospects

The definition of Biology as a modern Science is due to Darwin and his ability to see the overwhelming importance of sexual reproduction in animals and plants, as a mystery of Nature, as well as to grasp the selectiveness of females from what he knew about emotions. The origin of sex differences in humans has been attributed by social scientists to socialization and cultural influences, and only more recently biological influences, especially sex hormones are being considered (Geary, 2006).

The 21^st^ century sees a revolution in Biology that stays, however, inside the specialized scientific community. It refers to the question of “gene expression,” which is changing completely the naive idea of DNA definition of everything “by principle.” DNA expression depends on the chemical exterior circumstances, and this represents almost a revival of a modern Lamarckism based on “chemical ambient.”

A meeting held in United Kingdom brought together evolutionary and biomedical researchers working on early-life effects, and demonstrated that experiences during early development can trigger developmental switches that shape anatomy, physiology and behavior for a lifetime, while potentially also affecting future generations (Kuijper et al., 2019). It is time to focus on embryologic development, abandoned in the modern synthesis for ideological reasons.

Acceptance of biological basis for human behavior is coming together with acceptance of flexibility and even “culture” on non-humans, particularly the anthropoid primates (Whiten, 2005). This corresponds also to a movement in direction of bringing humans closer to nature, and a possible integration of the Nature vs. Culture dichotomy.

The previous items discussed objective arguments for efficient reproduction and infant survival, based on physical properties of hominoid hairs. It is time now to face Darwin’s intriguing idea, considered really “dangerous,” of esthetic reasons for female choice in sexual reproduction. Many papers exist on this question, but I mention only a recent article (Ryan, 2021) that discusses interesting aspects of this question, starting from fitness advantages through mate choice, followed by sensory ecology and signal design, investigations of neural circuits, neurogenetics, and neurochemistry underlying sexual attraction and more recently human studies in psychophysics, behavioral economics, and neuroaesthetics of its higher-order mechanisms, and finally cognitive ecology. This corresponds indeed to an integration of Nature with Culture.

The influence of culture is of course immense in most aspects of human societies, but there is a field connected to the body of females where biology crosses with esthetics and beauty: the integumentary system, which besides the skin also includes the hair, nails, and exocrine glands. This complex includes health, medicine and commerce since antiquity, as can be seen for instance in the Project Archeology (Ludlow, 2020). Darwin was able to foreseen this complex in connections with sexual reproduction in Life.

From the genetic front, four structural protein genes associated with the epidermal basement membrane zone or elastic fibers in the dermis were identified and were expressed significantly greater in humans than in non-human great apes (Arakawa et al., 2019) and may enhance the strength of adhesion between the epidermis and dermis in human skin. But it is necessary to focus also skin properties.

The subject of skin color has been extensively studied by Jablonski showing that the primary biological role of human skin pigmentation is protection against ultraviolet radiation (UVR). A recent review article (Jablonski, 2021) shows that the evolution of human skin pigmentation involved the interactions of genetic, environmental, and cultural variables, related to UVR and also vitamin D. Jablonski mentions indigenous groups with female pigmentation lighter than male, and suggests the importance of increased cutaneous vitamin D production potential, and therefore lighter skin, in order to facilitate absorption and redistribution of dietary calcium to the developing fetus and nursing neonate. Selection related to sexual reproduction, attractiveness coming as a consequence.

It is time now to focus on elastic properties of the human skin, much more complex than the study of hair, but it is necessary to enter now on this subject also. Scientific studies on the skin of rat started in the 80’s, extension to the human skin came on the 90’s and an article from this period gives an initial approach to the experimental techniques (Edwards and Marks, 1995). Many *in vitro* and *in vivo* experiments have been performed and it can be clearly stated that skin shows extremely complicated viscoelastic behavior. It could be concluded that the strength and elastic properties of skin are determined by the collagen content and visco elastic properties arise largely from the fluid (blood, lymph) and water content of skin. The large amount of results with many different techniques awaited some modeling for interpretation. But up to now there has been no convergence in the scientific literature on efficient modeling of human skin under all the experimental conditions.

The next step was given in 2021 by articles connected to the industry of beauty: A model of 3D dermal microtissue to study the skin’s elastic properties—Premium Beauty News, easily find on the internet. Therefore it seems to be approaching the crossing foreseen by Darwin between necessity and esthetics.

It is still necessary to join human skin with the skin of the Great Apes. Human skin was compared with skin of other primates by Montagna (1972, 1982, 1985). Human skin is known to be thicker than that of furred mammals and there are similarities with skin of chimpanzees and gorillas (Montagna, 1985).

It is possible now to advance in this subject with my own contribution. From the obtained estimation of 1 kg/cm^2^ as the safe limit for pressure on hominoid skin (Amaral, 2008) previously mentioned and shown in Figure 6, it can be now made here a very crude estimate of the Young modulus of elasticity of Hominoid skin as 0.1 MPa.

The result of 0.1 MPa here estimated for hominoid skin is not easily compared with existing studies of human skin elasticity, which varies with region of the body, age, sex, and other circumstances. But evidence the necessity of this type of investigation in order to clarify Human Evolution.

Human skin is the largest organ in the human body, responsible for our unique properties of sensibility and tactile body communication. It defines a unique interface between our inside and the outside, and such interface makes us Humans.

The essential legacy of Darwin on Sexual Selection remains on his focus on Human Skin, and the differences in relation to the skin of other primates. Darwin’s ideas shall be rescued as selection due to interactions between individuals within the species, besides interactions with the external ambient. Qualitative arguments on positive natural selection (sometimes called anecdotal evidence) may be meaningful from their clear function. It is time to recognize that both harmony and strength are pursued by Nature for their own intrinsic values, not for an ultimate finality.

## Author Contributions

The author confirms being the sole contributor of this work and has approved it for publication.

## Conflict of Interest

The author declares that the research was conducted in the absence of any commercial or financial relationships that could be construed as a potential conflict of interest.

## Publisher’s Note

All claims expressed in this article are solely those of the authors and do not necessarily represent those of their affiliated organizations, or those of the publisher, the editors and the reviewers. Any product that may be evaluated in this article, or claim that may be made by its manufacturer, is not guaranteed or endorsed by the publisher.
